# Intravenous Leiomyomatosis: The Importance of an Early Diagnosis

**DOI:** 10.7759/cureus.84486

**Published:** 2025-05-20

**Authors:** Tânia Boto, Catarina Magalhães Silva, Sofia Morgado Oliveira, Mariana Pereira Pinto, António Ribeiro Vieira, Carolina Piloto Lemos, Diana Neves Correia, Joana Albuquerque, Isabela Queimadela, Filipa Garcez Fernandes

**Affiliations:** 1 Family and Community Medicine, Unidade de Saúde Familiar (USF) Infante D. Henrique (Family Health Unit Infante D. Henrique), Viseu, PRT; 2 Family and Community Medicine, Unidade de Saúde Familiar (USF) Leiria Nascente (Family Health Unit Leiria Nascente), Leiria, PRT; 3 Family and Community Medicine, Unidade de Saúde Familiar (USF) Cidade Jardim (Family Health Unit Cidade Jardim), Viseu, PRT

**Keywords:** clinical awareness, diagnosis, intravenous leiomyomatosis, primary healthcare services, surgical resection

## Abstract

Intravenous leiomyomatosis (IVL) is a rare benign smooth muscle neoplasm characterized by intravascular proliferation, often extending from the uterus to the inferior vena cava and right heart. Usually identified incidentally, IVL may present with nonspecific symptoms, including abdominal distension, weight loss, and easy bruising, particularly in women. This case report describes a 47-year-old Caucasian female who presented with progressive abdominal enlargement and other nonspecific symptoms. Imaging revealed a giant tumor occupying the abdominal and pelvic cavities, and exploratory laparotomy was proposed. The surgical intervention involved total hysterectomy and bilateral salpingo-oophorectomy, revealing IVL with extensive involvement of the uterine and para-adnexal tissues. Post-operative recovery was uneventful, and no recurrence was noted over three years of follow-up. This case highlights the importance of recognizing IVL's potential presentations and advocating for timely diagnosis and intervention. Awareness among healthcare professionals is crucial to improve outcomes in patients with this rare entity, which poses potentially severe complications.

## Introduction

Intravenous leiomyomatosis (IVL) is a rare benign smooth muscle neoplasm that proliferates intravascularly and can extend from the uterus to the inferior vena cava and right heart, occasionally causing severe complications. IVL is often discovered incidentally, but it can present with symptoms such as abdominal distension, weight loss, easy bruising, uterine bleeding, or right heart failure, especially in women in their fifth decade [1-3]. Complete surgical resection, including hysterectomy and salpingo-oophorectomy, is the recommended treatment. Options for unresectable or recurrent cases are limited [2]. This case report aims to alert healthcare professionals to the presentation of IVL, emphasizing the importance of early recognition of warning signs.

## Case presentation

A 47-year-old Caucasian female was scheduled for a family planning appointment with her family doctor to perform cervical cancer screening. She reports no gynecological or other complaints. Her personal history reveals iron deficiency anemia, a simple left ovarian cyst (diagnosed in 2015), and a subserous uterine fibroid (diagnosed in 2016). The patient had missed a follow-up visit to the gynecology outpatient clinic. Her gynecologic and obstetric history reveals menarche at 10 years old, regular catamenia, and gravida 2, para 2 (G2P2). She was treated with oral iron and used a hormonal intrauterine device (IUD). She reports no smoking or alcohol habits.

On gynecological examination, the vulva, vagina, and cervix were unremarkable. Cervical cancer screening was performed without complications. On physical examination, the abdomen presented as globular, dull on percussion, and painless on superficial palpation, but very tense, rendering deep palpation impossible, and a tense hypogastric region was noted. When actively questioned, the patient reported a progressive increase in abdominal volume over the last four months, associated with weight loss (3 kg, which represents 5% of her weight), anorexia, tiredness, and easy bruising.

**Figure 1:**
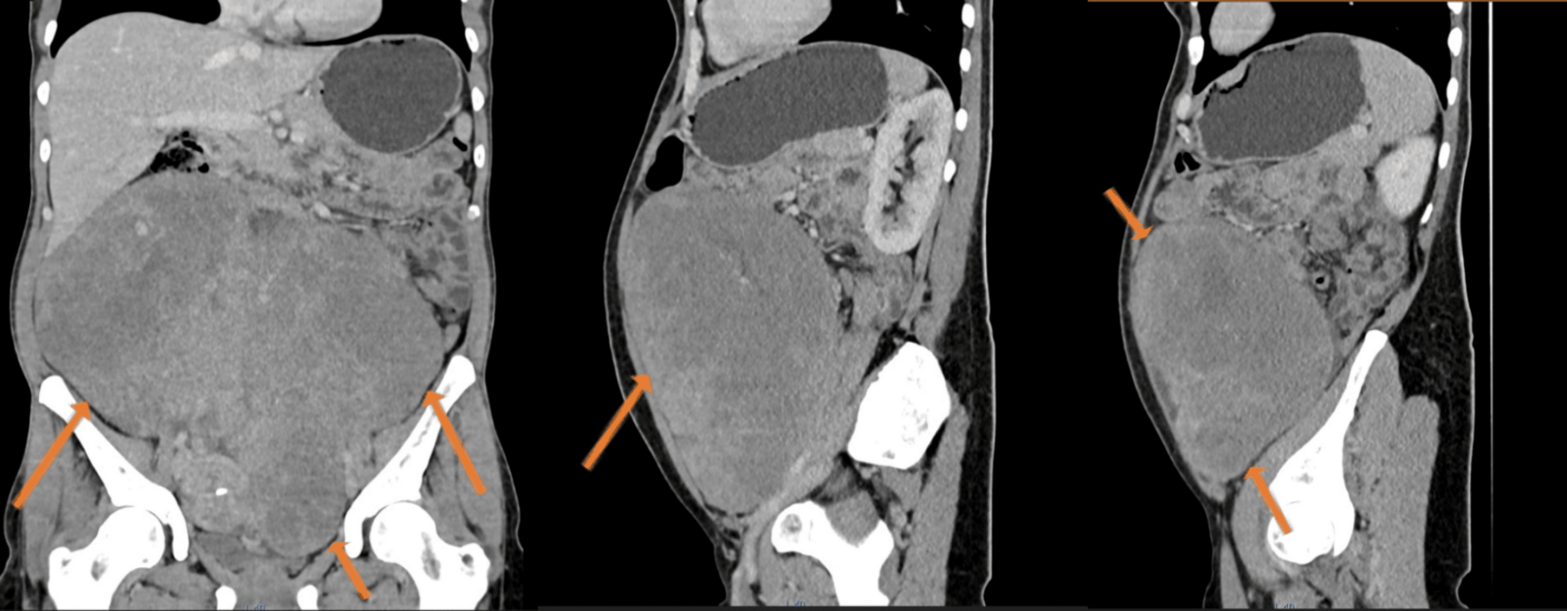
Images of an abdominopelvic CT scan that shows a giant mass (limited by arrows), which measures 30 x 28 x 16 cm, in the abdominal and pelvic cavity.

Abdominal and gynecological ultrasounds were requested but were inconclusive due to poor visualization. Therefore, the patient underwent an abdominopelvic computerized tomography (CT) scan (Figure 1), which revealed "a giant tumor, practically occupying the entire abdominal and pelvic cavity, solid and well-defined. It enhances after intravenous contrast, presents several areas of necrosis, and measures 30 x 28 x 16 cm. No adenopathies or peritoneal carcinomatosis referenced.” No relevant laboratory results. Negative cervical cancer screening.

The family doctor referred the patient to the gynecology emergency service due to a high suspicion of a gynecological tumor. When evaluated by a gynecologist, the mass had a hard-elastic consistency and showed solidarity with the uterus (upon touch). These findings were suggestive of an ovarian mass, and exploratory laparotomy was then proposed with priority scheduling (risk of ovarian malignancy algorithm (ROMA): 1.5%: low risk).

The patient underwent exploratory laparotomy, which revealed a large, regular left adnexal formation measuring approximately 30 cm. Peritoneal lavage, total hysterectomy, and bilateral adnexectomy were performed. Later that night, a reoperation was needed due to hemoperitoneum, with drainage of approximately 1-1.5 liters of bloody content from the abdominal cavity, with no obvious bleeding point detected, just a discreet towel-like "drooling" from the vaginal dome. The patient had a favorable evolution and was discharged on day eight.

The left adnexectomy surgical specimen revealed a “para-adnexal nodular formation measuring 27 x 21 x 16 cm, with a pinkish and lobulated surface, weighing 3683 g. The para-adnexal mass consists of white/pink, elastic, and fasciculated tissue, sometimes showing well-defined nodules, sometimes with areas of a varied appearance, partly yellow and partly congestive, involving multiple vascular structures in an area of ​​23 x 17 cm, focally coinciding with the surface." The right adnexectomy surgical specimen revealed “vascular dilation in the para-adnexal vessels due to the endovascular proliferation of leiomyomas.” Lastly, the uterine surgical specimen revealed “several leiomyomatosis lesions of moderate cellularity, without atypia or mitotic activity, with scattered vessels with thickened walls, and endovascular growth mainly in the background.” The cytological results of the peritoneal lavage were negative for neoplastic cells.

Anatomopathology diagnosed IVL involving the uterus and para-adnexal tissue bilaterally. An echocardiogram and a thoracic-abdominal-pelvic CT scan were performed to investigate the involvement of abdominal and thoracic vessels, which revealed no abnormalities. 

The patient was then discharged from the outpatient clinic since there was no indication for follow-up. Additionally, she had a menopause consultation and was informed that she should not take hormone replacement therapy. More than three years after the diagnosis of IVL, the patient remains without evidence of recurrence.

## Discussion

IVL is a rare entity, and approximately 300 cases have been reported to date [4,5]. It can originate from the wall of a blood vessel or a uterine leiomyoma [6]. The smooth muscle cells that cause IVL express progesterone and estrogen receptors, and this condition is associated with high estrogen levels [4,6].

The risk factors identified for developing this condition include the presence of uterine fibroids, previous surgeries such as hysterectomy or myomectomy, and a prior diagnosis of IVL [5].

Diagnosis of this entity can be challenging, as patients may remain asymptomatic even until the intravenous extension stage. When symptomatic, it can mimic a uterine fibroid, presenting as a pelvic mass or abnormal uterine bleeding. If there is an invasion of the heart chambers, it can result in chest pain, dyspnea, or, in the most severe cases, cardiac arrest [5,6].

In this case report, the patient presented with nonspecific symptoms of increased abdominal circumference, weight loss, anorexia, and easy bruising, which made the diagnosis even more challenging. After an initial inconclusive study, the CT scan revealed a large solid mass that occupied almost the entire abdominal and pelvic cavity, with areas of necrosis, compatible with an ovarian mass.

Despite being a benign condition, treatment, regardless of the stage, is surgical, consisting of total hysterectomy, bilateral salpingo-oophorectomy, and resection of any extra-gynecologic masses. The prognosis is usually favorable, as it is a slow-growing neoplasm [6].

## Conclusions

IVL is a rare entity but can have significant health consequences. Nonspecific symptoms can delay the diagnosis and, consequently, timely therapeutic decisions. For this reason, the family doctor must be attentive to and value ​​the signs and symptoms described by patients. Combined with a thorough clinical history, it is also important to consider conducting complementary diagnostic tests that can assist in the differential diagnosis.

The publication of this case aims to raise awareness about possible manifestations of this rare entity, to enable the most timely diagnosis possible, and to allow for correct treatment, thus improving the prognosis.
